# The Role of PXR Genotype and Transporter Expression in the Placental Transport of Lopinavir in Mice

**DOI:** 10.3390/pharmaceutics9040049

**Published:** 2017-10-24

**Authors:** Sarabjit S. Gahir, Micheline Piquette-Miller

**Affiliations:** 1 Leslie Dan Faculty of Pharmacy, University of Toronto, 144 College Street, Toronto, ON M5S 3M2, Canada; sarabjit.gahir@gmail.com; 2 Reata Pharmaceuticals, Irving, TX 75063, USA

**Keywords:** antiretrovirals, transporters, P-glycoprotein, breast cancer resistance protein, multidrug resistance associated protein, gene regulation, protease inhibitor, Pregnane X Receptor, placenta, knockout mice

## Abstract

Lopinavir (LPV), an antiretroviral protease inhibitor frequently prescribed in HIV-positive pregnancies, is a substrate of Abcb1 and Abcc2. As differences in placental expression of these transporters were seen in Pregnane X Receptor (PXR) −/− mice, we examined the impact of placental transporter expression and fetal PXR genotype on the fetal accumulation of LPV. PXR +/− dams bearing PXR +/+, PXR +/−, and PXR −/− fetuses were generated by mating PXR +/− female mice with PXR +/− males. On gestational day 17, dams were administered 10 mg/kg LPV (i.v.) and sacrificed 30 min post injection. Concentrations of LPV in maternal plasma and fetal tissue were measured by LC-MS/MS, and transporter expression was determined by quantitative RT-PCR. As compared to the PXR +/+ fetal units, placental expression of Abcb1a, Abcc2, and Abcg2 mRNA were two- to three-fold higher in PXR −/− fetuses (*p* < 0.05). Two-fold higher fetal:maternal LPV concentration ratios were also seen in the PXR +/+ as compared to the PXR −/− fetuses (*p* < 0.05), and this significantly correlated to the placental expression of Abcb1a (*r* = 0.495; *p* < 0.005). Individual differences in the expression of placental transporters due to genetic or environmental factors can impact fetal exposure to their substrates.

## 1. Introduction

Globally, there are over 2 million children living with HIV and, according to UNAIDS, most of these infections were caused by mother-to-child transmission (MTCT) of the virus [1]. Employing a strategy of aggressive, proactive prophylaxis when managing HIV-seropositive pregnancies by the administration of Highly Active Anti-Retroviral Therapy (HAART) has brought the rate of vertical transmission down from around 25% in the absence of these interventions to less than 2% in North America [2,3]. However, little is known about the factors controlling the transplacental trafficking of highly potent antiretrovirals.

The placenta is the principle gateway between the maternal and fetal systems, regulating the exchange of both endogenous and exogenous molecules. This barrier site also performs a critical role in limiting the access of potentially toxic xenobiotics [4,5]. The presence of several ATP Binding Cassette (ABC) drug transporters, including P-glycoprotein (PGP/ABCB1), Multidrug Resistance Associated Proteins (MRP/ABCC), and the Breast Cancer Resistance Protein (BCRP/ABCG2) at the materno-placental interface, is believed to be involved in the extrusion of a wide spectrum of drugs including antiretrovirals [6,7,8,9]. Indeed, the fetal accumulation of the protease inhibitor saquinavir has been found to be elevated in PGP-deficient mice models [10].

Lopinavir (LPV) is currently a second-line protease inhibitor (PI) that is frequently used in managing HIV-positive pregnancies [11,12]. Several in vitro and in vivo studies have established the involvement of ABCB1 and ABCC2 in the transport of LPV during the processes of absorption and disposition [13,14,15,16,17]. Inhibition or deficiency of PGP, which is encoded by Abcb1a and Abcb1b in rodents, has been shown to increase the oral bioavailability of LPV in addition to increasing its concentrations in the central nervous system (CNS). The placental transfer of PIs such as LPV has been shown to be highly variable in patients, in that it has been reported that the fetal cord to maternal plasma concentrations for LPV range from 0.05 to 0.34 [18,19,20]. Given the established role of drug transporters in LPV transport, the variability in the placental expression of these transporters may play an important part in inter- or intra-subject variability in the placental transfer of LPV into fetal tissues.

The expression of many drug transporters in the placenta has been shown to vary throughout gestation. Reports indicate that levels of PGP decline while levels of BCRP increase as gestation progresses [21,22,23]. The situation is further complicated by reports of disease-mediated alterations of drug transporters. In a study comparing the placental expression of PGP in HIV-infected and uninfected women, dramatically higher PGP expression was seen in placental tissue obtained from HIV-positive patients [24]. Alterations in the expression of the key drug efflux transporters in the placenta could have a serious clinical impact. In the case of drugs such as antiretrovirals, a fine balance between efficacy and safety needs to be struck, given the interplay between drug concentration, viral load suppression, and MTCT on one hand, and the potential for fetal drug toxicity on the other. Thus, it is important to be able to predict how genetic, environmental, or pathophysiological influences impact fetal drug exposure.

Nuclear receptors such as the Pregnane X Receptor (PXR) are known to be key regulators of ABC drug transporters such as Abcb1, Abcc2, Abcc3, and Abcg2 [25,26,27,28]. It is well known that PXR is activated by endogenous steroidal hormones and their metabolites, the levels of which are dramatically elevated during pregnancy. PXR is also activated by a number of dietary components, herbal remedies, and clinically important drugs [29,30]. Genetic polymorphisms of PXR are associated with decreased expression of the target genes encoding ABCB1 and CYP3A4 in patients, resulting in altered clearance of drug substrates [31,32]. While PXR activation and subsequent transporter induction at the liver and blood brain barrier has been shown to alter the bio-availability and CNS accumulation of many therapeutic agents, its role in determining fetal drug disposition is unclear and largely unexplored.

We have previously demonstrated a tissue-specific role for PXR in mice [33]. Several PXR target genes (Abcb1a, Abcc1-3, and Abcg2) were found to be elevated in the placenta in PXR null mice, with dramatically higher levels of placental transporters in the PXR −/− mice as compared to the PXR +/+ mice. As the functional units of the placenta are almost entirely derived from fetal tissues, it is the fetal genotype that dictates the placental genotype and is therefore responsible for regulating the expression of placental transporters [34]. In this manner, alterations of fetal PXR genotype could provide us with a unique murine model with clear differences in placental transporter levels within the same dam. Therefore, by breeding PXR heterozygotes (+/−), we generated a PXR +/− dam bearing PXR +/+, PXR +/−, and PXR −/− fetuses and placentas, allowing us to examine the impact of a range of placental transporter expression on substrate drug accumulation in the fetal units while maintaining a similar maternal physiological environment. This strategy enabled us to examine the relative contribution of placental transporters on LPV disposition without confounding maternal influences. Using this model, we explored the role of fetal PXR genotype and placental drug transporter expression on the fetal accumulation of LPV.

## 2. Materials and Methods

### 2.1. Animals and Experimental Design

All animal studies were in accordance with the guidelines of the Canadian Council of Animal Care. PXR heterozygote (+/−) animals were obtained by crossing PXR −/− females with PXR +/+ males. PXR wild type (+/+) C57/BL6 mice were purchased from Charles River Canada (Montreal, QC, Canada). The PXR knockout (−/−) C57/BL6 mice were obtained with approval from Dr. Steven Kliewer (University of Texas, Southwestern Medical Center, Dallas, TX, USA) as described previously [35]. For the purpose of obtaining timed pregnancies, the PXR +/− male mice were paired overnight with PXR +/− females, and the male removed the following morning contingent to observance of a vaginal plug. On gestational day (GD) 17, pregnant PXR +/− animals were administered 10 mg/kg LPV intravenously (i.v.) via tail vein (Lopinavir; USP, Rockville, MD, USA). LPV was dissolved in ethanol:propylene glycol: 5% dextrose solution (2:4:4 ratio). Animals were sacrificed at 30 min post injection and maternal plasma and fetal tissues were collected for analysis. Previously, we found that maximal fetal accumulation occurs at 30 min after LPV i.v. administration in healthy rats [36] and pilot studies in PXR +/− mice established that this time point was optimal in detecting LPV in both maternal and fetal samples. The plasma was stored at −20 °C, while the fetal and placental tissues were snap frozen and stored at −80 °C until analysis. The fetal units were genotyped for PXR by PCR from DNA isolated from placenta and visualizing the PCR products on a 2% agarose gel. The PXR genotype did not impact fetal survival nor fetal weight (PXR +/+: 0.72 ± 0.05 g; PXR +/−: 0.69 ± 0.02 g; PXR −/−: 0.75 ± 0.06 g).

### 2.2. Analysis of Transporter mRNA Expression

Methods for RNA isolation, cDNA synthesis, qRT-PCR, and primer sequences have been described previously [35]. Briefly, total RNA was extracted from placental tissue using the QuickPrep RNA extraction kit (Amersham Biosciences Inc., Piscataway, NJ, USA). RNA was quantified on a NanoDrop ND-1000 spectrophotometer (Thermo Fisher Scientific, Waltham, MA, USA) and then reverse-transcribed to cDNA by use of a first-strand cDNA synthesis kit (Fermentas, Burlington, ON, Canada) according to manufacturer’s protocol. qRT-PCR quantification of Abcb1a (Mdr1a), Abcc2 (Mrp2), and Abcg2 (Bcrp) mRNA were carried out by qPCR using Roche LightCycler technology with the LC FastStart DNA Master SYBR Green I Kit (Roche, Laval, QC, Canada). Oligonucleotides for previously reported primer sequences were synthesized at The Hospital for Sick Children (DNA Synthesis Centre, Toronto, ON, Canada). All transcript levels were normalized to the housekeeping gene, cyclophilin, using the efficiency-corrected ∆Cq qPCR method, and the ratios are presented as the percentage of control values. Normalization to either Gapdh or 18S rRNA was found to give comparable results, and cyclophilin levels were not significantly different between genotypes. Abcb1b (Mdr1b) was very poorly expressed in the placenta of our mice, with expression in most samples being below the detectable limit. As a result, Abcb1b mRNA levels are not reported.

### 2.3. Lopinavir LC-MS/MS Analysis

LPV concentrations in maternal plasma and fetal tissue samples were quantified using LC-MS/MS as previously described [36]. Briefly, maternal plasma and fetal tissue samples were thawed to room temperature. Tissue samples were homogenized in glass tubes with deionized water. Then, 100 μL of plasma or fetal tissue homogenates were added into tubes containing the internal standard, ritonavir (USP, Rockville, MD, USA). Sample extraction was performed using liquid-liquid extraction. Briefly, 50 µL of 500 mM sodium carbonate was mixed with the samples followed by adding 1.2 mL of hexane/ethyl acetate (1:1 *v*/*v*). The mixture was then vortexed for 2 min, centrifuged at 21,000 *g* for 15 min at 4 °C, and the organic layer (700 µL) was transferred to clean vials, evaporated under nitrogen gas, and reconstituted in 200 µL of 80% methanol. The final extracts were aliquoted into autosampler vials. Unless otherwise noted, all chemical reagents were purchased from Sigma-Aldrich (Oakville, ON, Canada).

The LC-MS/MS system employed a CTC PAL autosampler unit (LEAP Technologies, Carrboro, NC, USA) with an Agilent 1100 series pump (Agilent Technologies, Santa Carla, CA, USA). Elution was achieved using a 50 mm × 4.6 mm, 5 μm Lichrosorb RP-8 column (Phenomenex, Torrance, CA, USA) with a mobile phase of 20:80 parts of 0.1% formic acid to 80% methanol (flow rate 0.700 mL/min). MS/MS was performed with an API 4000 triple quadrupole MS equipped with a TurboIonSpray source and was set to the positive reaction monitoring mode (AB Sciex, Concord, ON, Canada). MRM transitions for LPV were *m*/*z* 629.3 to *m*/*z* 447.3 and for ritonavir, the internal standard, were *m*/*z* 721.3 to *m*/*z* 268, with the source temperature set to 500 °C. Analyst software version 1.4.2 was used (Applied Biosystems/MDS Sciex) for the analysis and quantification of LPV. The lower limit of LPV detection was <3 ng/mL and the lower limit of quantification was <10 ng/mL.

### 2.4. Statistical Analysis

Data were analyzed using GraphPad Prism (GraphPad Software version 5.0c, San Diego, CA, USA). Statistical significance was determined by analysis of variance (ANOVA) and significance was set to *p* < 0.05. Results are expressed as means ± standard error (SE). Pearson correlation (*r*) was used to analyze the strength of linear relationships between fetal LPV accumulation and transporter expression levels, and both the Pearson correlation r and absolute *p*-values are reported.

## 3. Results

### 3.1. Impact of Fetal PXR Genotype on Transporter Expression

**Figure 1:**
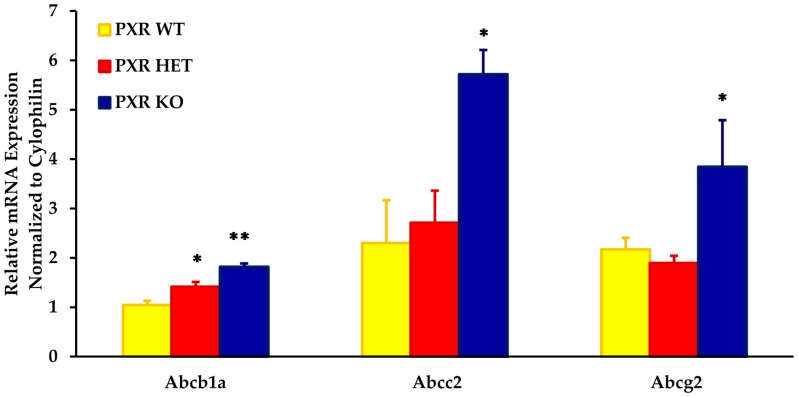
Effect of fetal genotype on basal mRNA expression of key placental transporters on GD 17. Fetal genotypes are PXR +/+ (PXR WT), PXR +/− (PXR HET), and PXR −/− (PXR KO). Placental mRNA expression was measured using RT-PCR and normalized to cyclophilin. Values are presented as relative expression levels ± S.E. * *p* < 0.05; ** *p* < 0.01 compared to PXR WT. *n* = 10.

As compared to the PXR +/+ fetal units, the placental mRNA expression of Abcb1a, Abcc2, and Abcg2 was approximately two- to three-fold higher in the PXR −/− fetal units (Figure 1). The expression of these transporters was significantly different in placentas obtained from PXR −/− fetal units as compared to PXR +/+ fetal units (*p* < 0.05).

Intermediate levels of these transporters were seen in fetal units with the PXR +/− genotype. While levels of Abcb1a in the PXR +/− placentae were significantly different from the PXR +/+ fetal units (*p* < 0.05), levels were not significantly different from the PXR −/− units. This provided us with an animal model with varying placental expression of transporters within the same dam.

### 3.2. Impact of Fetal PXR Genotype on Fetal Accumulation of LPV

**Figure 2:**
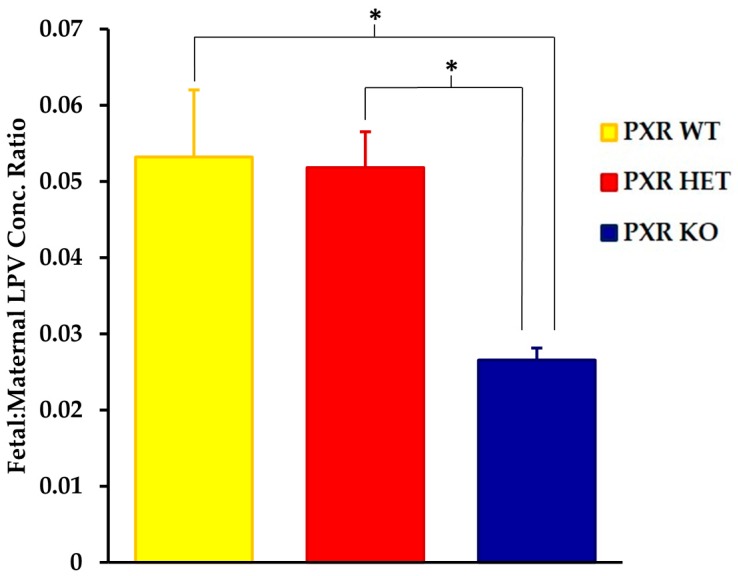
Effect of fetal genotype on LPV accumulation in fetal tissue. LPV concentration ratios were quantified by LC-MS/MS 30 min post-intravenous injection of LPV (10 mg/kg) as stated in the Materials and Methods section. Concentration ratios were calculated as the concentration ratio of LPV in fetal homogenate per unit weight (ng/g) to maternal plasma concentrations (ng/mL). Values are given as mean ratios ± S.E. * *p* < 0.05. *n* = 10.

We saw significant differences in the fetal tissue accumulation of LPV between the PXR +/+ and PXR −/− fetal units (Figure 2). The fetal tissue:maternal plasma LPV concentration was approximately 50% lower in the PXR −/− fetuses as compared to the PXR +/+ units (*p* < 0.05). There was, however, no statistically significant differences in LPV accumulation between the PXR +/+ and PXR +/− fetuses.

### 3.3. Relationship of Fetal Drug Accumulation and Transporter Expression

**Figure 3:**
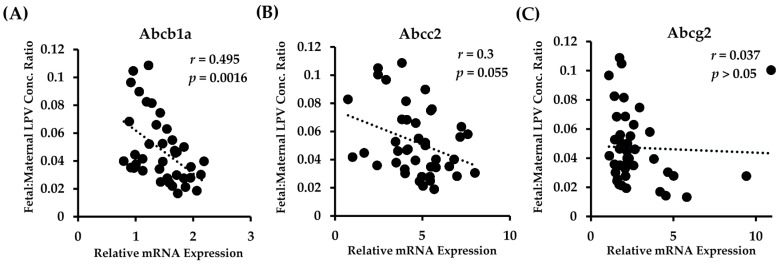
Relationship between fetal accumulation of LPV and placental expression of (**A**) Abcb1a; (**B**) Abcc2; and (**C**) Abcg2 in all fetal PXR genotypes. Fetal accumulation is represented as the LPV concentration of fetal homogenate (ng/g) relative to maternal plasma concentration (ng/mL). Relative placental mRNA expression was determined as described in the Materials and Methods section. *n* = 39.

We observed a highly significant correlation between the placental mRNA expression of Abcb1a in all fetal genotypes and the fetal accumulation of LPV (Figure 3A). There was a clear association of lower fetal tissue:maternal plasma concentration ratios with increased Abcb1a expression. A linear regression best fit the data, and the equation describing the data was *y* = 15.38*x* + 3.3 (*p* = 0.0016).

There was a strong trend with Abcc2 in that higher transcript levels of Abcc2 tended to be associated with lower fetal tissue:maternal plasma concentration ratios (Figure 3B). However, this association failed to reach significance (*p* = 0.055). The data were best described with a linear regression fit, with the equation of the line of best fit being *y* = 1.82*x* + 14. Of note, we found that the placental mRNA expression of Abcc2 was highly and significantly correlated to the expression of Abcb1a (*r* = 0.54; *p* = 0.0007). No significant correlation (linear or polynomial) between Abcg2 expression and LPV fetal accumulation was detected (Figure 3C).

## 4. Discussion

Many studies to date have reported low and highly variable placental transfer of protease inhibitors at delivery. While numerous in vitro and ex vivo studies have explored the role of placental transporters in drug transport, relatively little is still known about their genetic, environmental, and pathophysiological regulation, or the impact that changes in transporter expression have on fetal drug exposure. Our findings demonstrate that fetal PXR genotype affects the placental expression of drug transporters with a corresponding effect on fetal drug exposure. In this study, we found that the expression of three key ABC drug transporters, Abcb1a, Abcc2, and Abcg2, were significantly lower in fetal units with the PXR +/+ genotype as compared to the PXR −/− genotype. Additionally, we found a corresponding change in the accumulation of LPV in that the fetal-maternal concentration ratios in fetuses with the PXR +/+ genotype was approximately two times higher than those seen in the PXR −/− fetuses. These findings indicate that PXR genotypes that influence placental expression of transporters can also affect the fetal exposure to their substrates.

In vitro and in vivo studies have previously demonstrated that LPV is transported by PGP, which is primarily encoded by Abcb1a in murine placenta. Our model provided us with a four-fold range in the placental expression of Abcb1a between fetal units of different genotypes while maintaining a similar maternal environment. With an increase in the transcription level of placental Abcb1a, we saw a distinct decrease in the fetal accumulation of LPV. Moreover, the fetal accumulation of LPV was significantly correlated to Abcb1a expression, illustrating the important role of placental PGP on fetal exposure to LPV. These findings are in line with an ex vivo perfused human placental study that demonstrated that the inhibition of PGP increased the fetal to maternal transfer of LPV up to 10-fold [37]. Pathophysiological changes in placental Abcb1 expression were also found to significantly alter maternal-fetal transfer of LPV in rat models of infection and gestational diabetes [36,38].

We observed a very low and frequently undetectable placental expression of Abcb1b in all PXR genotypes. While PGP is encoded by Abcb1a and Abcb1b in rodents, it has been previously demonstrated that Abcb1a is the functional isoform in murine placenta. Studies in Abcb1a- and Abcb1b-deficient mice demonstrated that fetal exposure of PGP substrates such as digoxin were dependent on Abcb1a but not Abcb1b expression [39]. Moreover, only fetuses from dams with reduced Abcb1a expression (Abcb1a −/− or +/−) were susceptible to avermectin-induced cleft palate teratogenicity, despite uniform Abcb1b placental expression [40]. This highlights Abcb1a as the vital and functional isoform of PGP in mouse placenta.

The efflux transporter ABCC2, which is localized on the apical surface of the placenta, has also been shown to mediate placental transfer of its drug substrates such as talinolol [41]. Fetal:maternal concentration ratios of LPV tended to be lower, albeit non-significant, in fetal units with higher placental levels of Abcc2, suggesting possible involvement in the placental transport of LPV. However, the contribution of ABCC2 to the in vivo disposition of LPV is not clearly known. ABCC2 has been shown to transport LPV at low concentrations in vitro in transfected cell lines [14]. However, using knockout mice models, van Waterschoot et al. (2010) demonstrated that PGP but not ABCC2 significantly influenced the oral bioavailability of LPV [17]. As we found a strong association between the expression of Abcb1a and Abcc2, it is likely that the trend between Abcc2 expression and LPV fetal accumulation was due to the fact that placentas that expressed higher levels of Abcc2 also expressed higher levels of Abcb1a as a function of the PXR genotype, rather than implicating an active role of ABCC2 in placental LPV transport. While ABCG2 is highly expressed in the placenta and plays a key role in limiting maternal-fetal transport of potentially toxic xenobiotics, LPV is not a substrate of ABCG2 [14,42]. In line with previous data, we did not observe a significant association between the fetal accumulation of LPV and Abcg2 expression. However, LPV is a potent inhibitor of ABCG2 [43]. Therefore, given the importance of ABCG2 in fetal protection and its broad range of drug substrates, the potential for drug-drug interactions should be further explored. This is particularly important when considering the clinical scenario where pregnant HIV-positive patients receive numerous antiviral agents.

Interestingly, while we found significant differences in the placental expression of transporters between the PXR +/+ and PXR −/− fetal genotypes, these differences were not as great as those seen in our previous study [33]. For instance, while we previously saw a 12-fold higher expression of Abcb1a in the placenta of PXR −/− dams as compared to PXR +/+ dams, only a two-fold difference was seen in the placental expression between the PXR +/+ and PXR −/− fetuses. This may be due to the fact that while all three genotypes were generated within a single PXR +/− mother in this study, the previous study used homozygote PXR mating pairs to generate the three PXR genotypes (i.e., PXR +/+ parents to generate PXR +/+ fetus, PXR −/− parents to generate −/− fetus, and PXR −/− and PXR +/+ mating pair to generate PXR +/− fetus). Thus, the maternal environment to which the different PXR genotypic placentas were exposed were unique and likely had an impact on the expression of PXR target genes. It is well known that progesterone is an activator of PXR and levels of this steroid hormone increase throughout pregnancy. Moreover, PXR, along with other nuclear receptors, is believed to play a role in hormonal homeostasis and may subsequently regulate transporters through hormonal changes [44,45]. Therefore, differences in genotype as well as the physiological hormone environment may influence placental transporter expression in the different dams.

In conclusion, our data demonstrates the importance of PGP expression in limiting the fetal accumulation of an important antiretroviral agent. Hence inter-individual differences in the expression of placental transporters due to genetic or environmental factors can impact fetal exposure to their substrates. The situation is further confounded by the fact that expression may be further affected by pathophysiological changes due to HIV infection or other co-morbidities. Alterations can lead to either unexpected fetal accumulation or inadequate therapeutic levels, both resulting in poor fetal outcomes. The current model can be used to study the in vivo impact of placental transporter expression on the fetal exposure to a wide array of drugs, thus helping to bridge a critical knowledge gap in the area of drug usage in pregnancy.

## Abbreviations

ABC	ATP Binding Cassette
BCRP	Brest Cancer Resistance Protein
CNS	Central Nervous System
GD	Gestational Day
HAART	Highly Active Antiretroviral Therapy
HIV	Human Immunodeficiency Virus
I.V.	Intravenously
LC-MS/MS	Liquid Chromatography-Tandem Mass Spectrometry
LPV	Lopinavir
MRP	Multidrug Resistance Associated Protein
MTCT	Mother-to-Child Transmission
PI	Protease Inhibitor
PXR	Pregnane X Receptor
qRT-PCR	Quantitative Real Time Polymerase Chain Reaction
